# Engagement of persons with lived experience in research that uses linked administrative health data - the BC Provincial Overdose Cohort.

**DOI:** 10.23889/ijpds.v7i3.2100

**Published:** 2022-08-25

**Authors:** Amanda Slaunwhite, Heather Palis, Chloe Xavier, Roshni Desai, Bin Zhao, Wenqi Gan

**Affiliations:** 1 University of British Columbia; 2 BC Centre for Disease Control

Illicit drug overdose is a significant public health challenge in British Columbia (BC) that has been worsened by the COVID-19 pandemic. In 2021, 2224 persons died of overdose in BC– more than any year on record. The vast majority of overdose deaths are attributed to consumption of illicit substances and poisoning due to fentanyl. Since the 2016 emergency declaration, efforts have been made to create new data infrastructure that allows for comprehensive ascertainment of non-fatal and fatal overdose. The BC Provincial Overdose Cohort (BC-ODC) is a unique cohort that was created under public health order that includes all identified cases of illicit drug overdose in BC from January 1 2015-December 31, 2020 that is updated annually. With its unique data extracts, shared data governance structure and novel mandate, a critical component of stewarding the BC-ODC is engagement of persons with lived experience of substance use, overdose and/or incarceration. Two initiatives were launched in 2019 and 2022 to work with persons with lived experience to prioritize research that uses the BC-ODC data. Engagement of persons with lived experience of incarceration started in 2022 to inform projects that examined topics related to decriminalization, criminal legal system involvement (charges and convictions) and incarceration. This presentation will describe these initiatives to show the importance of peer involvement in research that uses linked administrative health data particularly when designing, interpreting and disseminating findings to reduce stigma towards persons who use substances.

